# Endogenous Omega (n)-3 Fatty Acids in Fat-1 Mice Attenuated Depression-Like Behavior, Imbalance between Microglial M1 and M2 Phenotypes, and Dysfunction of Neurotrophins Induced by Lipopolysaccharide Administration

**DOI:** 10.3390/nu10101351

**Published:** 2018-09-21

**Authors:** Minqing Gu, Yuyu Li, Haiting Tang, Cai Zhang, Wende Li, Yongping Zhang, Yajuan Li, Yuntao Zhao, Cai Song

**Affiliations:** 1 Research Institute for Marine Drug and Nutrition, College of Food Science and Technology, Guangdong Ocean University, Zhanjiang 524088, China; guminqingwawa@163.com (M.G.); YUYULI2016@163.com (Y.L.); THT19118@126.com (H.T.); zhangcai910206@163.com (C.Z.); zhangyp2015@163.com (Y.Z.); m13246531887@163.com (Y.L.); yuntaozhao@163.com (Y.Z.); 2 Shenzhen Institute of Guangdong Ocean University, Shenzhen 518120, China; 3 Guangdong Key Laboratory for Research and Development of Natural Drug, Guangdong Medical College, Zhanjiang 524023, China; lwd@gdlami.com; 4 Guangdong Key laboratory of Laboratory Animal, Guangdong Laboratory Animals Monitoring Institute, Guangzhou 510663, China; 5 Department of Psychology and Neuroscience, Dalhousie University, Halifax, NS B3H 4R2, Canada

**Keywords:** Fat-1 transgenic mice, n-3 fatty acids, microglial M1 and M2 phenotypes, neurotrophins, BDNF, depression

## Abstract

n-3 polyunsaturated fatty acids (PUFAs) have been reported to improve depression. However, PUFA purities, caloric content, and ratios in different diets may affect the results. By using Fat-1 mice which convert n-6 to n-3 PUFAs in the brain, this study further evaluated anti-depressant mechanisms of n-3 PUFAs in a lipopolysaccharide (LPS)-induced model. Adult male Fat-1 and wild-type (WT) mice were fed soybean oil diet for 8 weeks. Depression-like behaviors were measured 24 h after saline or LPS central administration. In WT littermates, LPS reduced sucrose intake, but increased immobility in forced-swimming and tail suspension tests. Microglial M1 phenotype CD11b expression and concentrations of interleukin (IL)-1β, tumor necrosis factor (TNF)-α, and IL-17 were elevated, while M2 phenotype-related IL-4, IL-10, and transforming growth factor (TGF)-β1 were decreased. LPS also reduced the expression of brain-derived neurotrophic factor (BDNF) and tyrosine receptor kinase B (Trk B), while increasing glial fibrillary acidic protein expression and pro-BDNF, p75, NO, and iNOS levels. In Fat-1 mice, LPS-induced behavioral changes were attenuated, which were associated with decreased pro-inflammatory cytokines and reversed changes in p75, NO, iNOS, and BDNF. Gas chromatography assay confirmed increased n-3 PUFA levels and n-3/n-6 ratios in the brains of Fat-1 mice. In conclusion, endogenous n-3 PUFAs may improve LPS-induced depression-like behavior through balancing M1 and M2-phenotypes and normalizing BDNF function.

## 1. Introduction

Inflammatory factors can stimulate the hypothalamic-pituitary-adrenal (HPA) axis to secrete glucocorticoids and activate glial cells to release proinflammatory mediators in the brain [1]. Both excessive production of glucocorticoids and proinflammatory cytokines can contribute to neuron apoptosis and dysfunction of neurotransmission and neurotrophins, thereby triggering depression [2]. There are two phenotypes of microglia, M1 and M2 [3]. The activated M1 phenotype expresses CD11b, CD68, and pro-inflammatory cytokines, including interleukin (IL)-1β, tumor-necrosis factor (TNF)-α, and IL-6, which have been extensively reported to induce oxidative stress, neuronal damage, neurotransmitter dysfunction, and depression-like behavior. By contrast, the activated M2 phenotype may up-regulate arginase (Arg)-1 and anti-inflammatory cytokines IL-10, IL-4, and transforming growth factor (TGF)-β1, which can reduce inflammation and protect neurons. Two microglial states can interact with astrocytes in the brain. The later can release a variety of neurotrophic factors, such as brain-derived neurotrophic factor (BDNF), glial-derived neurotrophic factor (GDNF), and fibroblast growth factor. These neurotrophic factors can nourish neurons, promote nerve growth, and maintain synaptic plasticity and transmission [4]. Studies from clinical investigation and animal experiments reported that increased depressive-like behavior was associated with a decrease in the total number of astrocytes in the anterior cortex of mice [5] and deficient BDNF in the hippocampus [6]. Thus, neurotrophin deficit has been hypothesized as a cause of depression [6].

To explore the role of neuroinflammation in depression, the model induced by intra-cerebroventricular (ICV) administration of lipopolysaccharides (LPS) has been popularly used due to several advantages over other models. It can first activate the HPA axis and microglia innate immune response [2]; secondly, it can significantly induce anxiety and depression-like behavior and impair memory [7]; thirdly, it can cause the dysfunction of monoamine neurotransmitters and induce serotonergic neuron death in the hippocampus and raphe nucleus [8]. All these changes induced by LPS are similar to those observed in depression.

As inflammation significantly contributes to the etiology of depression, anti-inflammatory treatment has become a new direction. Recent studies from our lab and others have shown that dietary supplementation with n-3 polyunsaturated fatty acids (PUFAs) can effectively improve depressive changes [9,10] due to their anti-inflammation and anti-oxidative stress properties, and cause up-regulation of neurotrophins such as BDNF [11]. The best understood mechanism by which n-3 PUFAs treat depression is by inhibiting n-6 fatty acid arachidonic acid (AA) from converting into eicosanoid (a precursor of pro-inflammatory mediators) through cyclooxygenase and lipoxygenase pathways [12]. However, previous results of n-3 PUFA treatment for depression from both clinical investigations and animal models were gained by oral administration in depressed patients or rodent models of depression. PUFA purities, caloric content, and ratios in different diets were varied. Moreover, n-3 PUFA metabolism and interactions with other food components in the digestive system are unknown. To eliminate the effects of potential confounding factors of n-3 diets and to further explore the mechanism by which n-3 PUFAs modulate microglial M1 and M2 phenotypes (astrocytes producing neurotrophin and receptor functions in depression), we utilized the transgenic Fat-1 mice, which convert n-6 PUFA to n-3 PUFA in the brain [13]. With Fat-1 mice, the present study tested the hypothesis that endogenously elevated brain n-3 PUFA concentrations can significantly attenuate LPS-induced depression-like behaviors through microglial activities, such as by inhibiting microglial M1 but enhancing the M2 phenotype, restoring astrocyte and neurotrophic function, and reducing proinflammatory cytokine production and apoptosis-related gene expression. These changes should be correlated with increased in n-3 PUFA production in the brain.

## 2. Materials and Methods

### 2.1. Animals

Heterozygous transgenic Fat-1 mice, which express the *Caenorhabditis elegans* Fat-1 gene, were generated as described previously [13] and backcrossed onto a C57BL/6J background. The Fat-1 mice carry a Fat-1 transgene from the roundworm *Caenorhabditis elegans*, enabling them to endogenously convert n-6 to n-3 PUFAs. The animals attain high tissue levels of n-3 PUFAs even when fed an n-6 PUFA-rich diet [13]. The presence of the Fat-1 gene in each mouse was confirmed both by genotyping and brain tissue fatty acid analysis profile (see below). In this study, Fat-1 transgenic mice were mated with wild type C57BL/6 female mice to obtain Fat-1 positive C57BL/6 mice (Fat-1) and Fat-1 negative C57BL/6 mice (WT). Fat-1 and WT animals were fed with diet 10% soybean oil and kept under pathogen-free conditions in standard cages in temperature- and humidity-controlled conditions with a 12-h light/dark cycle. All animal care and handling procedures were conducted in compliance with the National Institutes of Health Guide for Care and Use of the laboratory animals and approved by the Local Bioethics Committee (Guangdong Ocean University, Zhanjiang, China; document number: SYXK2014-0053).

### 2.2. Genotyping

DNA was extracted from approximately 2–3 mm of the mouse toe by Mouse Tail SuperDirect™ PCR kit (FOREGENE, Chengdu, China). The primers used for the Fat-1 gene were forward: 5′-CTGCACCACGCCTTCACCAACC-3′ and reverse: 5′-CACAGCAGCAGATTCCAGAGATT-3′. PCR was performed on Super cycler (Kyratec, Mansfield, Australia). The PCR reaction was performed at 95 °C for 15 min, followed by 30 cycles of 94 °C for 30 s, 62 °C for 30 s, 72 °C for 60 s, and a final extension at 72 °C for 10 min. Amplified PCR products were analyzed on 1% agarose gels and amplified bands were visualized by the automatic gel system (Tanon 3500, Shanghai, China).

### 2.3. ICV Saline or LPS Injection

**Figure 1.**
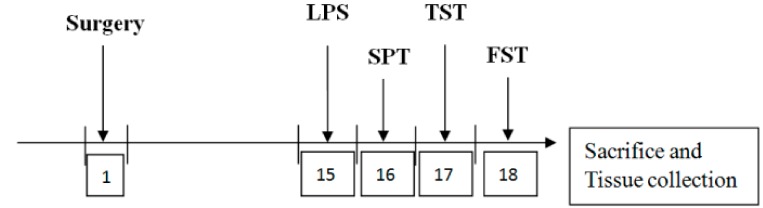
Timeline of the experimental procedures. SPT: sucrose preference test; TST: tail suspension test; FST: forced swimming test; LPS: lipopolysaccharide.

Here, 48 males (aged 2 months) were divided into four groups: wild-type mice/saline (WT/saline); Fat-1 mice/saline (Fat-1/saline); wild-type mice/LPS (WT/LPS); and Fat-1 mice/LPS (Fat-1/LPS). Mice were injected with 1 μL of saline or 3 μg/μL LPS (*Escherichia coli* 0127:B8, Sigma-Aldrich, St. Louis, USA) into the right lateral ventricle. Briefly, each mouse was placed in a stereotaxic apparatus (David Kopf, Instruments, Tujunga, CA, USA) after being anesthetized with salbutamol hydrochloride (15 mg/kg, intraperitoneal, IP)), zolazepam (15 mg/kg, IP, and xylazine hydrochloride (23 mg/kg, IP). Guide cannulae (for ICV administration) were inserted stereotaxically into the right lateral ventricle (AP = −0.6 mm, ML = 1.2 mm, DV= −1.8 mm). Cannulae were secured to the skull with screws and dental acrylic. 14 days after the surgery, mice were injected with saline/LPS into the lateral ventricle. Behavioral tests were performed in mice after 24 h of treatment with ICV injection. Following behavioral tests, the hippocampus was removed and stored with the rest of the brain in 80 °C for the following experiment. Figure 1 presents a timeline of the experimental procedures.

### 2.4. Behavioral Tests

#### 2.4.1. Sucrose Preference Test

The sucrose preference test [14] was modified to determine the anhedonic state of mice. Prior to beginning the test, mice were habituated to the presence of two drinking bottles (1% sucrose) for 24 h, and one of the bottles of 1% sucrose was substituted with fresh water for 24 h. Then, 24-h food and water deprivation was applied. The sucrose preference test was measured by liquid consumption (1% sucrose or water) for 4 h and calculated according to the following formula: SP = sucrose intake/(sucrose intake + water intake) × 100%.

#### 2.4.2. Tail Suspension Test

The tail suspension test was employed to estimate stress associated with depression in rodents as previously described [15]. Briefly, mice with a medical tape placed 1 cm from the tip of the tail were hung on the suspension test instrument holder for 5 min, approximately 20 cm away from the ground. The immobility time was recorded by an infrared camera.

#### 2.4.3. Forced Swimming Test

The forced swimming test was performed by the method previously described [16]. Briefly, mice were forced to swim in an open cylindrical container (diameter 10 cm, height 30 cm) containing 20 cm of fresh water maintained at 25 ± 1 °C. Water in the cylinder was changed after each test. The immobility time was recorded during 5 min testing period.

### 2.5. Fatty Acid Analysis in the Brain

The tissue was flash frozen in liquid nitrogen and stored at −80 °C. The composition and concentration of PUFAs were determined by GC as described previously [17]. Briefly, brain tissue was homogenized with chloroform/methanol solution (2/1). After vortexing, NaCl solution was added. The bottom phase was collected after centrifugation at 500 rpm for 20 min. The extracts were dried by nitrogen and incubated with sulfuric acid methanol solution and methylene chloride solution in a water bath at 100 °C for 1 h. After cooling, hexane and H_2_O were added and centrifuged at 500 rpm for 2 min. The hexane phase containing fatty acid methyl ester (FAME) lipids was dried by nitrogen and collected for GC analysis. The FAME was analyzed by GC using a Trace GC Ultra (Thermo Fisher Scientific, Waltham, MA, USA) equipped with a flame ionization detector. The PUFA peak was identified by comparing retention times with external FAME standard mixtures (Nu-Chek-Prep, 606, Main St. Elysian, MN, USA) and docosapentaenoic acid (DPA) PUFA standard (Nu-Chek-Prep, U-101-M, Main St. Elysian, MN, USA). The n-3 and n-6 PUFA contents in the brain of Fat-1 mice and WT littermates were measured. The n-3 PUFAs include α-linolenic acid (ALA, 18:3 n-3), eicosapentaenoic acid (EPA, 20:5 n-3), n-3 docosapentaenoic acid (n-3 DPA, 22:5 n-3), and docosahexaenoic acid (DHA, 22:6 n-3), while n-6 PUFAs include linolcic acid (LA, 18:2 n-6), γ-linolenic acid (GLA, 18:3 n-6), dinomo-γ-linolenic acid (DGLA, 20:3 n-6), and arachidonic acid (AA, 20:4 n-6).

### 2.6. ELISA Assays

The concentrations of IL-1β, IL-4, IL-10, IL-13, IL-17, TGF-β1, TNF-α, iNOS, and NO in the hippocampus were determined by the commercial enzyme linked immunosorbent assay (ELISA) kits from Beijing Dongge Biotechnology Co., Ltd. (Beijing, China) in accordance with the manufacturer’s protocols.

### 2.7. Real-Time PCR

The total RNA was extracted from hippocampal tissues by trizol (Life, USA) and was transcribed into cDNA by a commercial RT-PCR kit (Vazyme, Nanjing, China) according to the manufacturer’s instructions. The primer sequences of CD11b, BDNF, Trk B, p75, and glial fibrillary acidic protein (GFAP) listed in Table 1 were purchased from Sangon Biotech (Shanghai, China). The real-time PCR reaction was run on a CFX Connect™ Real-Time system (Bio-Rad laboratories, Hercules, CA, USA), with the process of an incubation for 3 min at 95 °C, followed by 40 cycles of 95 °C for 5 s and then 60 °C for 30 s. The melting curve was generated for the determination of primer specificity and identity. Gene expression levels were quantified by normalizing Ct values of target genes to Ct values of the reference gene (β-actin) with the ΔΔ*C*_t_ method [18].

### 2.8. Western Blot

The hippocampal tissues were homogenized in the RIPA buffer, and then spun down at 13,000 rpm for 10 min. After adding sample buffer and boiling, the supernatant was collected and loaded to SDS PAGE gels. After running the gels, the proteins were transferred to PVDF membranes and blocked for 1 h in 5% non-fat dry milk. The following antibodies and concentrations were used over night at 4 °C: CD11b (Abcam; 1:1000, Cambridge, MA, USA), BDNF (Abcam; 1:1000; Cambridge, USA), pro-BDNF (Santa Cruz; 1:200; Santa Cruz, CA, USA), Trk B (Abcam; 1:1000; Cambridge, MA, USA), GFAP (Santa Cruz; 1:1000; Santa Cruz, CA, USA), and p75 (Santa Cruz; 1:1000; Santa Cruz, CA, USA). The peroxidase-conjugated secondary antibodies were detected using an enhanced chemiluminescence (ECL) (Millipore Corp., Billerica, MA, USA). Densitometric analysis of the immunoreactive bands was performed using a chemiluminescence system (Tanon 5200, Shanghai, China). All target proteins were quantified by normalizing them to β-actin re-probed on the same membrane and then calculated as a percentage of the control group.

### 2.9. Statistical Analysis

All data were presented as mean ± SEM and analyzed by IBM SPSS 19.0 software. Statistical analyses for behavioral, PUFA profile, ELISA data and mRNA expression were performed by two-way ANOVA within the genotype (WT versus Fat-1) and model (saline versus LPS). When *p* < 0.05 was shown by the ANOVA, the difference between groups was analyzed by Bonferroni *post hoc* test, while the mRNA expression was analyzed by Fisher’s LSD *post hoc* test. The statistical significance of protein expression was calculated by a Student’s unpaired *t*-tests. Non-parametric data, such as NO, were analyzed by Kruskal–Wallis followed by the Dunn–Bonferroni *post hoc* test. *p* < 0.05 was considered statistically significant.

## 3. Results

### 3.1. Fatty Acid Profile in the Brain

As shown by Table 2, two-way ANOVA analysis indicated that significant impact of the genotype on the concentrations of DHA (F_1,42_ = 37.533, *p* < 0.001), EPA (F_1,42_ = 10.349, *p* < 0.01), DGLA (F_1,39_ = 5.794, *p* < 0.05), total n-3 PUFA (F_1,39_ = 30.435, *p* < 0.001), n-3/n-6 ratio (F_1,39_ = 13.566, *p* < 0.001), and AA/DHA ratio (F_1,39_ = 26.001, *p* < 0.001). The Bonferroni *post hoc* test further revealed that DHA and EPA levels were significantly higher in Fat-1 mice than those in WT littermates (DHA: *p* < 0.001, EPA: *p* < 0.05), while the DGLA level was lower in Fat-1 than those in WT mice (*p* = 0.05). Overall, subtotal n-3 PUFA concentrations were increased (*p* < 0.01), and the AA/DHA ratio was decreased (*p* < 0.01), while subtotal n-6 PUFA concentrations and AA/EPA ratio were unchanged in Fat-1 mice when compared to WT mice.

### 3.2. Depressive-Like Behaviors Induced by LPS Were Attenuated in the Fat-1 Mice

**Figure 2.**
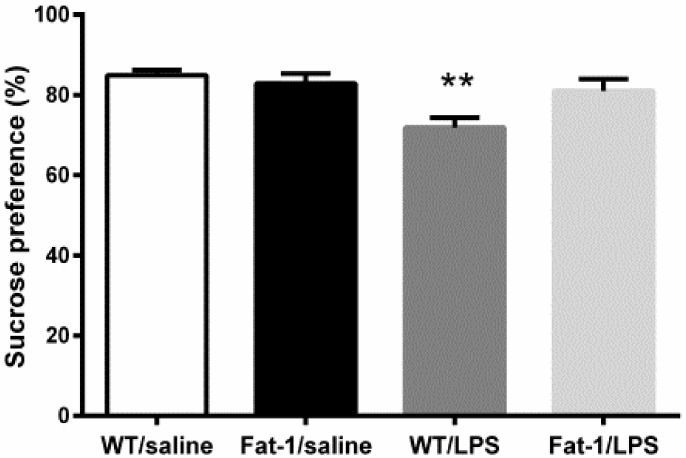
LPS decreased sucrose preference in wild-type mice, which was not reversed in Fat-1 mice. ** *p* < 0.01 vs. WT/saline. LPS: lipopolysaccharides; WT: Wild-type.

As shown by Figure 2, two-way ANOVA analysis showed a significant effect of LPS (F_1,30_ = 9.806, *p* < 0.01) and interaction between the genotype and LPS (F_1,30_ = 5.446, *p* < 0.05). In correlation with this statistical result, the Bonferroni *post hoc* test revealed that the sucrose consumption was decreased LPS injected mice (*p* < 0.01), which was not reversed in Fat-1 mice (*p* = 0.094).

**Figure 3.**
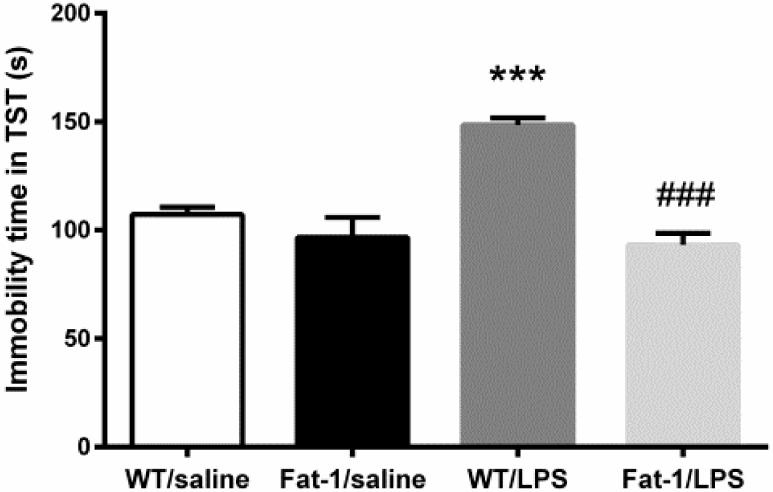
LPS increased immobility time in tail suspension test (TST), which was significantly reversed in Fat-1 mice. *** *p* < 0.001 vs. WT/saline; ^###^ *p* < 0.001 vs. WT/LPS. LPS: lipopolysaccharides; WT: Wild-type.

As shown in Figure 3, two-way ANOVA analysis suggested a significant impact of LPS on immobility time in the tail suspension test (F_1,39_ = 11.278, *p* < 0.01). Meanwhile, the interaction between LPS and genotype was also significant (F_1,39_ = 15.862, *p* < 0.001). The Bonferroni *post hoc* test further revealed that the immobility time was increased in WT mice after LPS injection (*p* < 0.001), which was significantly reversed in Fat-1 mice (*p* < 0.001).

**Figure 4.**
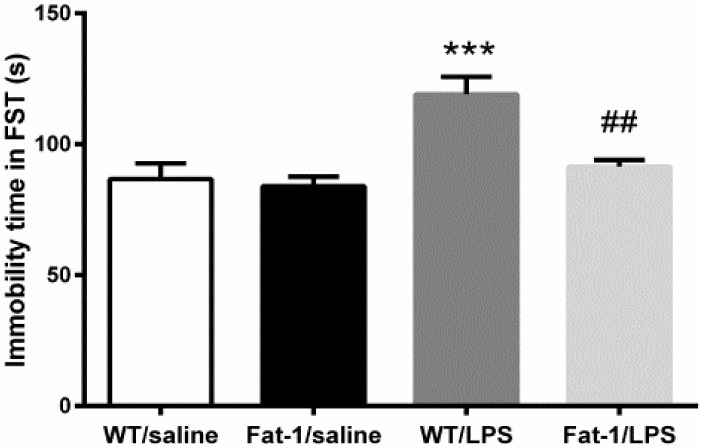
LPS increased immobility time in forced-swimming test (FST), which was attenuated in Fat-1 mice. *** *p* < 0.001 vs. WT/saline; ^##^ *p* < 0.01 vs. WT/LPS. LPS: lipopolysaccharides; WT: Wild-type.

Similarly, as shown by two-way ANOVA, there was a significant effect of LPS (F_1,30_ = 16.376, *p* < 0.001) and interaction between the genotype and LPS (F_1,30_ = 6.435, *p* < 0.05). Then, the Bonferroni *post hoc* test further revealed that LPS significantly increased the immobility time as compared to control group in the forced-swimming test (FST) (*p* < 0.001). However, immobility time induced by LPS was significantly reversed in Fat-1 mice when compared to WT mice (*p* < 0.01) (Figure 4).

### 3.3. LPS-Induced M1 Polarization and Cytokine Changes in the Hippocampus

**Figure 5.**
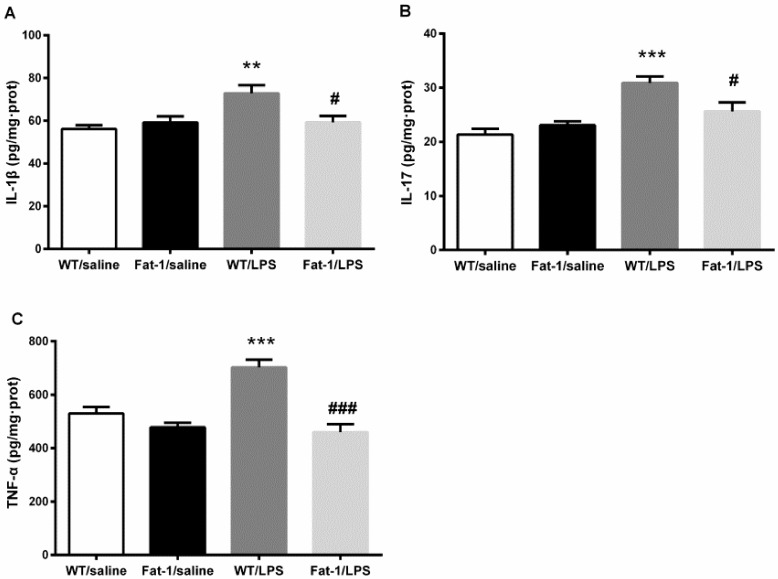
LPS up-regulated interleukin (IL)-1β, IL-17, and tumor necrosis factor (TNF)-α concentration significantly in WT littermates. These changes were markedly attenuated in Fat-1 mice. (**A**) IL-1β; (**B**) IL-17; (**C**) TNF-α. ** *p* < 0.01, *** *p* < 0.001vs. WT/saline; ^#^ *p* < 0.05, ^###^ *p* < 0.001 vs. WT/LPS. LPS: lipopolysaccharides; WT: Wild-type.

Two-way ANOVA indicated that LPS significantly affect the concentration of IL-1β (F_1,41_ = 3.141, *p* < 0.01), TNF-α (F_1,41_ = 33.678, *p* < 0.001), and IL-17 (F_1,40_ = 23.773, *p* < 0.001) in the hippocampus, while the genotype also affected TNF-α (F_1,41_ = 33.678, *p* < 0.001) concentrations. Meanwhile, there was an interaction between LPS and the genotype in IL-1β (F_1,41_ = 7.762, *p* < 0.01), TNF-α (F_1,41_ = 14.06, *p* < 0.001), and IL-17 (F_1,40_ = 7.891, *p* < 0.01). The Bonferroni *post hoc* test showed that IL-1β (*p* < 0.01), TNF-α (*p* < 0.001), and IL-17 (*p* < 0.001) concentrations were significantly up-regulated after LPS injection in WT littermates compared to the control. These changes were markedly attenuated in Fat-1 mice for IL-1β (*p* < 0.05), TNF-α (*p* < 0.05), and IL-17 (*p* < 0.001) (Figure 5).

### 3.4. The Effect of LPS on M2 Polarization and Related Factors in the Hippocampus

**Figure 6.**
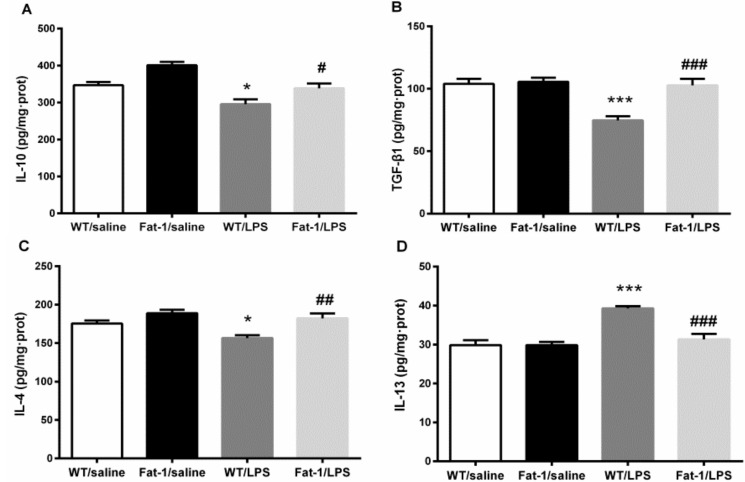
LPS decreased the concentration of IL-10, IL-4 and transforming growth factor (TGF)-β1, but increased IL-13. These abnormalities were attenuated significantly in Fat-1 mice for IL-10, IL-4, TGF-β1, and IL-13. (**A**) IL-10; (**B**) TGF-β1; (**C**) IL-4; (**D**) IL-13. * *p* < 0.05, *** *p* < 0.001 vs. WT/saline; ^#^ *p* < 0.05, ^##^ *p* < 0.01, ^###^ *p* < 0.001 vs. WT/LPS. LPS: lipopolysaccharides; WT: Wild-type.

Two-way ANOVA indicated that LPS significantly changed the concentration of IL-10 (F_1,31_ = 26.253, *p* < 0.001), TGF-β1 (F_1,31_ = 16.595, *p* < 0.001), IL-4 (F_1,31_ = 7.652, *p* < 0.01), and IL-13 (F_1,41_ = 24.594, *p* < 0.001), and the genotype affected the concentrations of IL-10 (F_1,31_ = 19.131, *p* < 0.001), TGF-β1 (F_1,31_ = 14.181, *p* = 0.001), IL-4 (F_1,31_ = 17.416, *p* < 0.001), and IL-13 (F_1,41_ = 13.092, *p* = 0.001). Meanwhile, there was an interaction between LPS and the genotype in the concentration of IL-13 (F_1,41_ = 12.970, *p* < 0.001) and TGF-β1 (F_1,31_ = 11.374, *p* < 0.01). In correlation with this statistical result, the Bonferroni *post hoc* test revealed that LPS decreased the concentration of IL-10 (*p* < 0.05), IL-4 (*p* < 0.05) and TGF-β1 (*p* < 0.001), but increased IL-13 compared to the control (*p* < 0.001). These abnormalities were attenuated significantly in Fat-1 mice for IL-10 (*p* = 0.05), IL-4 (*p* < 0.01), TGF-β1 (*p* < 0.001), and IL-13 (*p* < 0.001) (Figure 6).

### 3.5. Oxidative Stress-Related Nitric Oxide Enzyme iNOS and NO Levels

**Figure 7.**
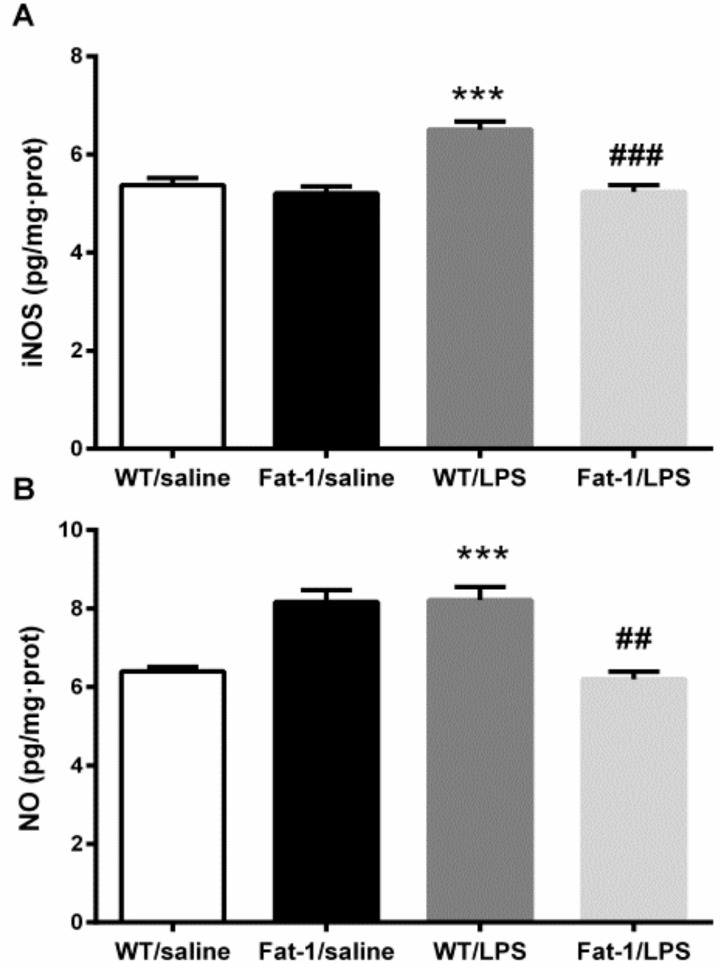
LPS increased iNOS and NO concentration. These increases were attenuated in Fat-1 mice signally. (**A**) iNOS; (**B**) NO. *** *p* < 0.001 vs. WT/saline; ^##^ *p* < 0.01, ^###^ *p* < 0.001 vs. WT/LPS. LPS: lipopolysaccharides; WT: Wild-type.

Two-way ANOVA indicated that LPS significantly changed the concentration of iNOS (F_1,41_ = 15.229, *p* < 0.001), and the genotype also affected iNOS (F_1,41_ = 23.687, *p* < 0.001) levels. The interaction between LPS and the genotype in iNOS was also significant (F_1,41_ = 13.912, *p* < 0.01). Furthermore, Kruskal–Wallis test showed that LPS and the genotype significantly influenced the NO expression (χ^2^(3) = 27.525, *p* < 0.001). Then, the *post hoc* test revealed that iNOS (*p* < 0.01) and NO (*p* < 0.001) were significantly increased by LPS. These increases were signally attenuated in Fat-1 mice (NO, *p* < 0.001; iNOS, *p* < 0.001) (Figure 7).

### 3.6. Expression of Neurotrophins and Their Receptors in the Hippocampus

**Figure 8.**
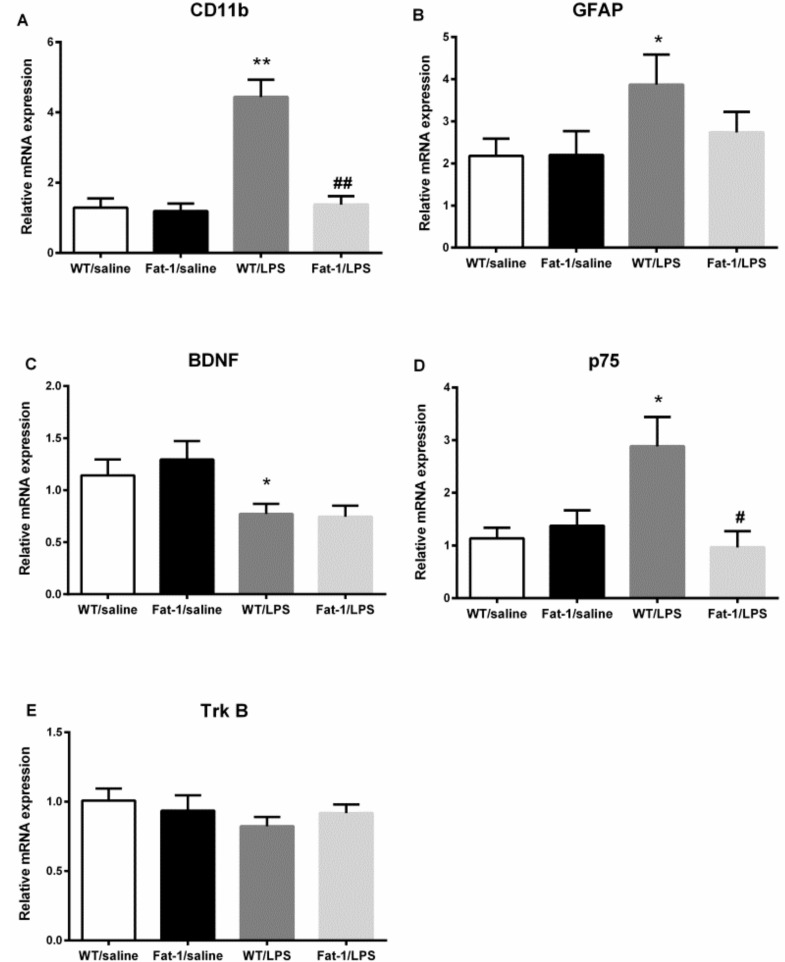
LPS up-regulated the mRNA expression of CD11b, GFAP, and p75, and down-regulated mRNA expression of BDNF in the LPS model. mRNA expression of CD11b and p75 were decreased in Fat-1 mice when compared with WT/LPS-challenged group. (**A**) CD11b; (**B**) GFAP; (**C**) BDNF; (**D**) p75; (**E**) Trk B. * *p* < 0.05, ** *p* < 0.01 vs. WT/saline; ^#^ *p* < 0.05, ^##^ *p* < 0.01 vs. WT/LPS. GFAP: glial fibrillary acidic protein; BDNF: brain-derived neurotrophic factor; LPS: lipopolysaccharides; WT: Wild-type.

Two-way ANOVA showed that LPS significantly influenced mRNA expression of CD11b (F_1,31_ = 28.389, *p* < 0.001), BDNF (F_1,60_ = 10.724, *p* < 0.01), p75 (F_1,26_ = 4.854, *p* < 0.05), and GFAP (F_1,24_ = 4.017, *p* < 0.05), but not Trk B. The interaction between LPS and Fat-1 significantly affected mRNA expression of CD11b (F_1,31_ = 22.441, *p* < 0.001) and p75 (F_1,26_ = 15.053, *p* < 0.01). In correlation with this statistical result, the Fisher’s LSD *post hoc* test revealed that LPS up-regulated the mRNA expression of CD11b (*p* < 0.001), p75 (*p* < 0.001), and GFAP (*p* < 0.05), down-regulated mRNA expression of BDNF (*p* = 0.05) in the LPS model. mRNA expression of CD11b (*p* < 0.001) and p75 (*p* < 0.001) were decreased in Fat-1 mice when compared to WT with LPS-challenged group. However, no significant change was found in Trk B expression (Figure 8).

**Figure 9.**
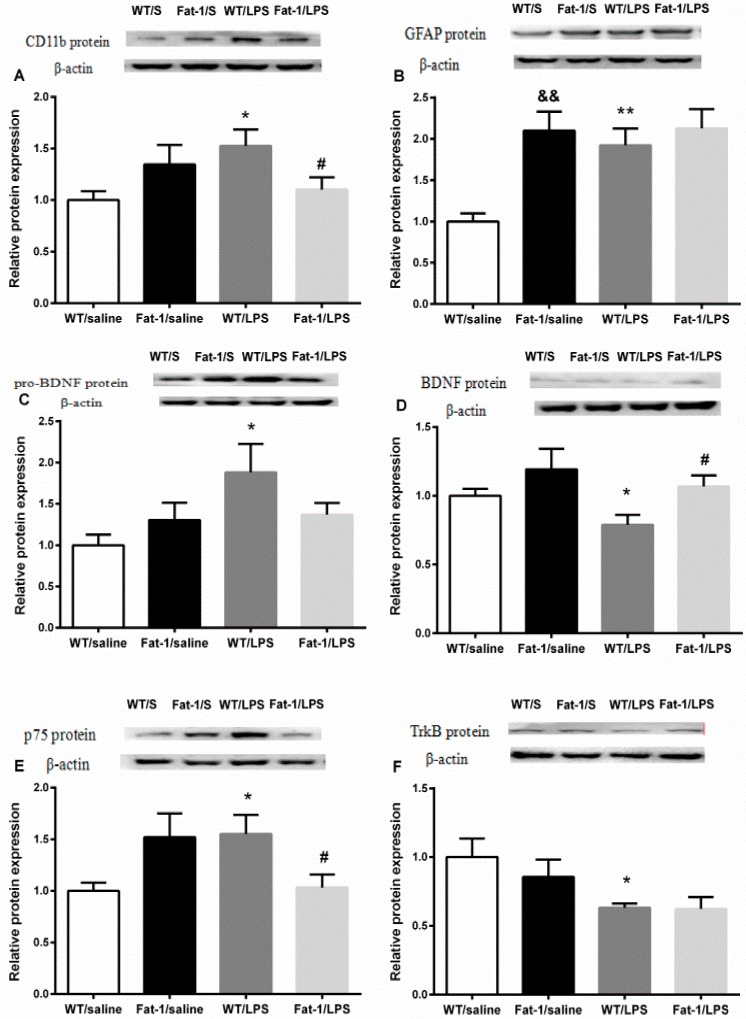
LPS significantly up-regulated the protein expression of CD11b, GFAP, p75, and pro-BDNF, but down-regulated protein expression of BDNF and Trk B in the animal model. The protein expression of GFAP was increased in Fat-1 mice when compared with wild-type mice. The rise in the protein expression of CD11b and p75 was prevented, and there was an up-regulation of BDNF level at the same time in Fat-1 mice. (**A**) CD11b; (**B**) GFAP; (**C**) pro-BDNF; (**D**) BDNF; (**E**) Trk B; (**F**) p75. * *p* < 0.05, ** *p* < 0.01 vs. WT/saline; ^#^ *p* < 0.05 vs. WT/LPS; ^&&^ *p* < 0.01 vs. Fat-1/saline. LPS: lipopolysaccharides; WT: Wild-type. S: saline.

The *t*-test showed that LPS significantly up-regulated the protein expression of CD11b (*p* < 0.05), GFAP (*p* < 0.01), p75 (*p* < 0.05), and pro-BDNF (*p* < 0.05), but down-regulated protein expression of BDNF (*p* < 0.05) and Trk B (*p* < 0.05) in the animal model. The protein expression of GFAP (*p* < 0.01) was also increased in Fat-1 mice when compared with those in wild-type mice. LPS-induced up-regulation in the protein expression of CD11b (*p* < 0.05) and p75 (*p* < 0.05), and down-regulation in BDNF was blocked in Fat-1 mice (*p* < 0.05). However, the changes of Trk B and pro-BDNF were not significantly reversed in Fat-1 mice (Figure 9).

## 4. Discussion

In the present study, LPS-induced depressive-like behaviors, such as decreased consumption of sucrose and increased immobility time in the forced-swimming test and tail suspension test in C57 mice, are similar to previous findings reported by others [19]. The behavioral changes may result from glial cell dysfunction in the brain, which we found in the present study. The functional polarization of neuroglia in depression, especially microglia and astrocyte interaction, has recently received more attention. Activated microglia have two polarizations, namely the M1 type (classical/proinflammatory activation) and M2 type (alternative/anti-inflammatory activation). The domination of M1 phenotype exaggerates neuroinflammation through releasing pro-inflammatory cytokines, such as IL-1β and TNF-α, but suppresses anti-inflammatory cytokines like IL-10 and TGF-β [3], which may contribute to the etiology of depression. On the contrary, effective antidepressant therapies can inhibit neuroinflammation by shifting M1 to M2 polarization [20,21]. In the present study, central administration of LPS caused increased M1 phenotype and suppressed M2, increasing IL-1β, IL-17, and TNF-α while decreasing IL-10, IL-4, and TGF-β1. However, IL-13, an anti-inflammatory cytokine from T-helper 2 in the periphery [22], was significantly increased after LPS injection in the hippocampus of wild-type mice. This change seems to be a puzzle. Previous studies reported that when rats were injected LPS (IP) and IL-13 (ICV) together, IL-13 potentiated LPS-induced depressive effects. With administration of IL-13 prior to LPS, the depressive effects of LPS could not be blocked [23]. Moreover, IL-13 can enhance COX-2 expression in activated microglia, thus exacerbating inflammation [24]. It has also been reported that the plasma level of IL-13 was significantly increased in mice after acute-foot shock [25]. In parallel, elevated serum levels of IL-13 were found in depressed patients [26]. Besides, increased iNOS and NO induced by IL-13 may also contribute to increased inflammatory response [27,28]. Taken together with our findings in this study, the function of IL-13 seems to be pro-inflammatory rather than anti-inflammatory in the brain. Given the bi-directional regulation of IL-13 in inflammation, we should pretreat mice with the IL-13 antibody or use L-NAME for NOS blockade in FAT1 mice in our further study.

Astrocytes, the other neuroglial cells, together with microglia, maintain central nervous system (CNS) homeostasis via regulate neuroinflammatory events and modulate neurotrophin function [29,30,31]. Acute neuroinflammation may activate, while chronic neuroinflammation may suppress astrocyte activity, as indicated by up-regulating and down-regulating GFAP expression, respectively [32,33]. Similarly, acute LPS-induced up-regulated GFAP was observed in the present study, which was associated with down-regulated BDNF and Trk B expression, but up-regulated pro-BDNF and p75 expression. Pro-BDNF binding with p75 neurotrophin receptor can induce neuronal atrophy and apoptosis, dendritic pruning, and long-term depression (LTD) [34]. By contrast, when binding to Trk B, BDNF can promote neurotransmission and neuroplasticity. However, BDNF can also lead to synaptic degeneration and even neuron apoptosis when binding to p75 [35]. Thus, the imbalance between BDNF and pro-BDNF can result in activation of p75 receptor function and neuronal apoptosis.

Prior studies, including our own, have demonstrated that behavioral abnormalities in depression are related to hyper-activation of microglia and their triggered neuroinflammation, which may induce astrocyte and neurotrophin dysfunction [36]. Except for these findings, the present study further provided new findings between microglial M1 and M2-phenotypes and pro- and mature BDNF function in the LPS-induced depression. The most important is that the present study for the first time showed that endogenous n-3 could reverse LPS-induced depression-like behavior from several aspects below.

First, endogenous n-3 PUFAs in Fat-1 mice can attenuate depressive-like behaviors, such as by reducing immobility time in both FST and TST, which was associated with a higher level of n-3 PUFAs in the brain. These findings confirmed previous studies in which diet enriched with n-3 PUFAs could improve depressive symptoms in depressed patients or in rodent models [10,37]. Previously, Marcelo et al. [38] found that fish oil supplementation could also reverse LPS-induced decrease in sucrose consumption. However, decreased sucrose preference was not significantly attenuated in Fat-1 mice in this study. Similar results were reported after n-3 PUFA treatment by others. For example, n-3 PUFAs did not reverse decreased sweet food intake in rats after CMS exposure [39]. A possible explanation was fish smell and gastrointestinal distress against sweet food intake. However, by using Fat-1 mice, the present study demonstrated that endogenous n-3 fatty acids that overcome fish smell and gastrointestinal distress cannot improve this anhedonic behavior in the depression model. In another chronic mild stress model of depression, neither fish oil- nor n-3 PUFA-enriched phospholipids supplementation could reverse the decreased sucrose intake in rats [40], a finding that is similar to our data. Because of limited literature, there is no clear explanation as to why n-3 PUFAs can improve most depression-like behaviors in several models of depression except for sucrose consumption. In the future, we should further explore the specific mechanism by which n-3 PUFAs affect anhedonic behavior, for example the function of dopaminergic system [41] and serotonin transporter protein expression [42] in Fat-1 mice, which are related to anhedonia in depression. The second major finding in the present study was to demonstrate that endogenous n-3 PUFAs can balance M1 and M2 phenotypes through the down-regulation of CD11b and reduction of M1-related inflammatory cytokine concentrations, and increased M2-related anti-inflammatory cytokines. A previous study showed that n-3 PUFAs can inhibit inflammation by inhibiting the release of TNF-α from primary microglia upon IFN-γ and myelin stimulation [43]. As mentioned above, a well-known anti-inflammatory mechanism is that n-3 PUFAs can decrease the precursor of inflammatory eicosanoids [44], and inhibit LPS–triggered NF-κB activation and translocation [45]. However, our data showed that AA concentration was unchanged in Fat-1 mice when compared to WT mice, even though subtotal n-3 PUFA concentrations and n-3/n-6 ratio were increased. Previously, Melanie et al. [46] reported similar results. These data may indicate that AA levels remain the same due to continued synthesis and conversion of LA from the n-6 PUFA diet.

In contrast to microglial M1, which induces inflammation, M2 exerts neuroprotective effects by secreting BDNF and anti-inflammatory cytokines. Moreover, microglia can interact with astrocytes to modulate the production of BDNF. For example, a mechanically-injured astrocyte-conditioned medium (ACM) could provoke microglial cells to promote the transcription, synthesis, and release of BDNF through p38-MAPK signaling pathway [47]. On the other hand, on incubation with a stimulated striatal microglia-conditioned medium (MCM), striatal astrocytes can be activated to express BDNF genes in return [48]. Thus, thirdly, the present study demonstrated that endogenous n-3 PUFAs in Fat-1 mice reversed LPS-induced abnormal BDNF function, such as the up-regulation of BDNF and down-regulation of p75, which may exert anti-depressant effects in the present study. These data confirmed other findings which showed a correlation between n-3 intake and peripheral BDNF levels after the intake of PUFAs as diet complement in clinical and experimental studies [49,50]. Conversely, the deficiency of n-3 in rats leads to a decrease of BDNF in the prefrontal cortex via a p38 MAPK-dependent mechanism [51]. Thus, supplementation of n-3 PUFAs can normalize BDNF levels and reduce oxidative damage in traumatic brain injury model of rats [52]. Fourthly, the present study found that endogenous PUFAs could reverse LPS-increased NO and iNOS concentrations in the hippocampus. Nitric oxide synthase-derived NO exerts a negative effect on the hippocampal neurogenesis [53]. Moreover, NOS inhibitors showed antidepressant-like properties under physiological conditions [54]. Similarly, n-3 PUFAs were found to suppress inflammation by inhibiting NF-κB/iNOS/NO signaling pathway activation, thus reducing iNOS mRNA synthesis and finally the production of NO [55,56].

## 5. Conclusions

In conclusion, by using Fat-1 mice to avoid the disadvantages of n-3 PUFAs in diets, the present study demonstrated that endogenous n-3 PUFAs could ameliorate depression-like behavior induced by ICV administration of LPS, which may be via an anti-inflammatory mechanism in Fat-1 mice because the M1 phenotype (increased CD11b expression) and pro-inflammatory cytokines IL-1β, IL-17, and TNF-α were inhibited, while the M2 phenotype and related anti-inflammatory cytokines IL-10, IL-4 and TGF-β1 were increased. These anti-inflammatory effects were accompanied by normalizing astrocyte function, shown through the decreased expression of p75, but increased BDNF, which may contribute to the improvement of depression-like changes.

## Figures and Tables

**Table 1.** Primer sequence used for real-time PCR.

Genes	Primer	Sequence
CD11b	Forward	5′-CCCATGACCTTCCAAGAGAA-3′
	Reverse	5′-AGAGGGCACCTGTCTGGTTA-3′
BDNF	Forward	5′-AGCTGAGCGTGTGTGACAGT-3′
	Reverse	5′-TCAGTTGGCCTTTGGATACC-3′
Trk B	Forward	5′-CACACACAGGGCTCCTTA-3′
	Reverse	5′-GTCAGCTCAAGCCAGACACA-3′
p75	Forward	5′-CCGATGCTCCTATGGCTACT-3′
	Reverse	5′-CTCTGGGCACTCTTCACACA-3′
GFAP	Forward	5′-GAAAGGTTGAATCGCTGGAG-3′
	Reverse	5′-GCCACTGCCTCGTATTGAGT-3′
â-actin	Forward	5′-GTCGTACCACTGGCATTGTG-3′
	Reverse	5′-CTCTCAGCTGTGGTGGTGAA-3′

BDNF: brain-derived neurotrophic factor; Trk B: tyrosine receptor kinase B; GFAP: glial fibrillary acidic protein.

**Table 2:** Fatty acid profile in mouse brain tissue.

Fatty Acids mg/g Tissue	Saline		LPS		Statistical Effects		
	WT *N* = 11	Fat-1 *N* = 11	WT *N* = 11	Fat-1 *N* = 10	LPS	Genotype	LPS × Genotype
EPA (20:5 n-3)	0.17 ± 0.03	0.33 ± 0.05 *	0.18 ± 0.04	0.27 ± 0.03	NS	<0.01	NS
DPA (22:5 n-3)	0.19 ± 0.04	0.27 ± 0.03	0.23 ± 0.04	0.29 ± 0.05	NS	NS	NS
DHA (22:6 n-3)	2.65 ± 0.07	3.08 ± 0.05 ***	2.76 ± 0.04	3.15 ± 0.10 ^##^	NS	<0.001	NS
Subtotal n-3	3.01 ± 0.11	3.59 ± 0.13 **	3.18 ± 0.69	3.71 ± 0.11 ^##^	NS	<0.001	NS
LA (18:2 n-6)	0.38 ± 0.03	0.52 ± 0.05	0.50 ± 0.06	0.48 ± 0.05	NS	NS	NS
DGLA (20:3 n-6)	0.12 ± 0.01	0.16 ± 0.02 *	0.015 ± 0.01	0.16 ± 0.01	NS	<0.05	NS
AA (20:4 n-6)	1.56 ± 0.05	1.55 ± 0.06	1.60 ± 0.04	1.58 ± 0.08	NS	NS	NS
Subtotal n-6	2.06 ± 0.05	2.21 ± 0.07	2.26 ± 0.07	2.19 ± 0.08	NS	NS	NS
n-3/n-6	1.46 ± 0.04	1.68 ± 0.06	1.42 ± 0.07	1.68 ± 0.03 ^#^	NS	<0.001	NS
AA/DHA	0.59 ± 0.01	0.51 ± 0.02 **	0.58 ± 0.01	0.50 ± 0.02 ^##^	NS	<0.001	NS

AA: arachidonic acid; DHA: docosahexaenoic acid; DGLA: dinomo-γ-linolenic acid; DPA: docosapentaenoic acid; EPA: eicosapentaenoic acid; LA: linolcic acid; LPS: lipopolysaccharides; NS: no significance. Data are mean ± SEM. * *p* < 0.05, ** *p* < 0.01, *** *p* < 0.001 vs. wild-type (WT)/saline; ^#^ *p* < 0.05, ^##^ *p* < 0.01 vs. WT/LPS.
